# Elevations in Liver Transaminases in COVID-19: (How) Are They Related?

**DOI:** 10.3389/fmed.2021.705247

**Published:** 2021-07-15

**Authors:** Henrique Pott-Junior, Natália Queiroz Prado Bittencourt, Silvana F. G. Chacha, Rafael Luís Luporini, Marcia Regina Cominetti, Fernanda de Freitas Anibal

**Affiliations:** ^1^Department of Medicine, Federal University of São Carlos, São Carlos, Brazil; ^2^Biotechnology Graduate Program, Federal University of São Carlos, São Carlos, Brazil; ^3^Department of Gerontology, Federal University of São Carlos, São Carlos, Brazil; ^4^Department of Biological Sciences Parasitology, Federal University of São Carlos, São Carlos, Brazil

**Keywords:** inflammation, interleukin-2, interleukin-10, lymphocytes, SARS-CoV-2, hepatitis, transaminases

## Abstract

Liver involvement in COVID-19 is not yet well-understood, but elevations in liver transaminases have been described to occur in 14–53% of the cases and are more frequently seen in severe disease. This cross-sectional study explored the relationship between the elevations in liver transaminases and inflammatory parameters in 209 adults with COVID-19. Demographic and clinical data, serum levels of inflammatory cytokines and liver aminotransferases were analyzed. Three groups were formed according to the liver transaminase abnormalities: (I) Normal transaminases, (II) Borderline transaminases elevation, and (III) Mild to severe transaminases elevation. Altered liver transaminases were directly related to disease severity, showing association with the NEWS2 score at admission and greater need for ICU or death. Moreover, higher levels of IL-2 and CRP were associated with borderline transaminases elevations, whereas higher levels of IL-10 and Neutrophil to Lymphocyte ratio were associated with mild to severe transaminases elevation. These results reinforce the importance of liver transaminases in patients with COVID-19 as a complementary marker for disease severity and also point to them as a parameter reflecting the continuous dynamics between viral infection and the immune response.

## Introduction

More than a year has passed since the severe acute respiratory syndrome coronavirus 2 (SARS-CoV-2) was first described, and several characteristics of the coronavirus disease (COVID-19) remain to be better explained. With more than 100 million reported cases of infection the COVID-19 reached pandemic status in March 2020, and up to date, no specific antiviral treatment has been proven to be effective against this disease, imposing an extensive burden on health care worldwide (1, 2).

During COVID-19, patients can be asymptomatic or present a wide range of clinical symptoms (3). Gastrointestinal symptoms have been described to occur up to 15% (4), and were attributed to viral cell invasion mediated by Angiotensin-Converting Enzyme 2 (ACE2) receptors at the enterocyte level (5). Although not fully understood, the involvement of the liver with elevated levels of hepatic enzymes in blood biochemistry tests has also been described to occur in 14–53% of the cases (6–10). Possible mechanisms that may be associated with liver damage during COVID-19 are (1) an immune-mediated inflammation; (2) a direct cytotoxic effect caused by viral replication of hepatocytes; (3) a drug-induced liver injury, including self-prescribing medications and those used for the treatment of COVID-19, such as remdesivir, tocilizumab, chloroquine; and (4) reactivation of previously existing liver diseases (11, 12).

Moreover, several studies have shown that abnormalities in liver biochemical tests are more frequent in severe cases of COVID-19 as compared to mild and moderate ones (4, 13). The association between abnormalities in liver biochemical tests and worse prognosis (5, 6, 10, 14–18) and mortality (19) has also been demonstrated. However, there is a lack of studies on the relationship between elevations of liver transaminases and inflammatory cytokines, seeking to understand these changes as a reflection of the ongoing dynamic between viral infection and the immune response. Evaluating the parameters involved in the severity of COVID-19 and their relationship with hepatic pathophysiology would provide relevant information for a better understanding of these changes, as well as the complications of this condition.

Additionally, it is still unclear whether the liver could be involved either as a direct target of the SARS-CoV-2 or secondary to the systematic changes promoted by the viral infection, mainly inflammation and cytokine release, immune response, and ischemia (17). Thus, in this study we aimed to further explore the relationship between the elevations of liver transaminases and inflammatory markers in adult patients with COVID-19.

## Methods

### Study Design, Participants, and Setting

This is a cross-sectional study conducted at the University Hospital of the Federal University of Sao Carlos (UFSCar) from June to December 2020. All patients admitted with more than 14 days from the illness onset were excluded from this study. The study was conducted according to the guidelines from the Declaration of Helsinki and all procedures involving research study participants were approved by the UFSCar's Research Ethics Committee (Number: 30184220.8.0000.5504). Written informed consent was obtained from all participants.

### Study Assessments

All patients were assessed at admission for demographic data, chronic comorbidities [Charlson Comorbidity Index (CCI)], and National Early Warning Score (NEWS) 2 for clinical deterioration. Laboratory data were also reviewed, and the plasma levels of inflammatory cytokines were assessed by commercial laboratory methods, as described further. Patients were categorized as mild, moderate, and severe according to the recommendations established by the WHO's COVID-19 Clinical management living guidance (20). All patients received standard of care treatment for COVID-19 as the latest recommendations on managing the disease, and other specific treatments were recorded, including dexamethasone (yes, no), low-weight molecular heparin (none, prophylactic, intermediary or therapeutic dose), and antibiotics (yes, no).

### Definitions of Liver Transaminase Abnormalities

A borderline elevation was defined as serum levels of alanine aminotransferases (ALT) and aspartate aminotransferases (AST) exceeding the upper limit of normal range (ULN) but less than two-fold the ULN. A mild to severe elevation was considered in patients with an increase of at least two-fold the ULN (21).

### Systemic Markers of Inflammation

Within the first 12 h of admission, venous blood was sampled from all patients to analyze systemic markers of inflammation. Samples were analyzed through flow cytometry (BD Accuri C6, BD Biosciences, San Diego, CA, USA), and serum cytokines (IL-2, IL-4, IL-6, IL-10, IFNγ, and TNFα) were measured with cytometric bead array human inflammation kit (BD™ CBA Human Th1/Th2 Cytokine Kit, BD Biosciences, San Diego, CA, USA). The procedure was conducted following the manufacturers' instructions and data were analyzed using FlowJo software (FlowJo LLC, Ashland, OR, USA).

### Statistical Analysis

Continuous data are presented as mean ± standard deviation or median [1st−3rd quartile] according to the Shapiro-Wilk normality test. Categorical variables are presented as counts (percentages). Comparisons between groups were performed using Kruskal-Wallis test followed by Bonferroni-Dunn's *post-hoc* test for continuous variables, and Pearson's Chi-squared test with Yates' continuity correction for categorical variables. Statistical significance was assessed at a two-sided *p*-value <0.05. All analyses were conducted using R version 4.0.3 (The R Foundation for Statistical Computing, Vienna, Austria) in R-Studio 1.3.1093 (RStudio Inc., Boston, USA).

## Results

A total of 209 patients consecutively admitted to hospital were included in this study. Table 1 depicts baseline characteristics of the cohort. Most of the subjects were male (56%) and aged mean 59.4 ± 18.4 years, ranging from 21 to 99 years old. The median Charlson Comorbidity Index was 2 [1st−3rd quartile, 0 4], and about a fifth (20%) of the subjects presented high comorbidity index (≥5). Most of the patients were not diabetic (76%) nor they had cardiovascular diseases (73%), nor were hypertensive (52%).

**Table 1:** Baseline characteristics of the cohort.

**Feature**	**Overall (*N* = 209)**
Age, years	59 [44, 74]
Female sex	92 (44)
Charlson comorbidity index (CCI)	2 [0, 4]
High comorbidity (CCI ≥ 5)	42 (20.1)
Comorbidities	
Arterial hypertension	100 (47.8)
Cardiovascular disease	35 (16.7)
Diabetes	50 (23.9)
NEWS2 on admission	4 [3, 6]
Disease severity	
Mild	22 (10.5)
Moderate	87 (41.6)
Severe	100 (47.8)
Time from symptom onset to hospital admission, days	7 [5, 10]
Gastrointestinal symptoms	
Diarrhea	40 (19.1)
Vomiting	20 (9.6)
Abdominal pain	16 (7.7)
Need for ICU admission during hospitalization	81 (38.8)
Laboratory tests on admission	
AST (U/L)	35 [24, 56]
ALT (U/L)	28 [18, 54]
Alkaline phosphatase (U/L)	66 [50, 84]
Gamma-glutamyl transferase (U/L)	54 [33, 121]
Total billirubin (mg/dL)	0.5 [0.4, 0.6]
Albumin (g/L)	
Lymphocyte count (×10^9^/L)	1.038 [0.67, 1.501]
Platelets (×10^9^/L)	224 [179, 275]
D-dimer (μg/ml)	880 [420, 1850]
Lactate dehydrogenase (U/L)	298 [219, 448]
Prescribed medications	
Low-molecular-weight heparin	
Prophylactic dose	74 (35.4)
Intermediary dose	43 (20.6)
Therapeutic dose	36 (17.2)
Glucocorticoids	136 (65.1)
Antibiotics	131 (62.7)

*Continuous data are presented as median [1st, 3rd quartile]. Categorical variables are presented as counts (percentages)*.

**Figure 1:**
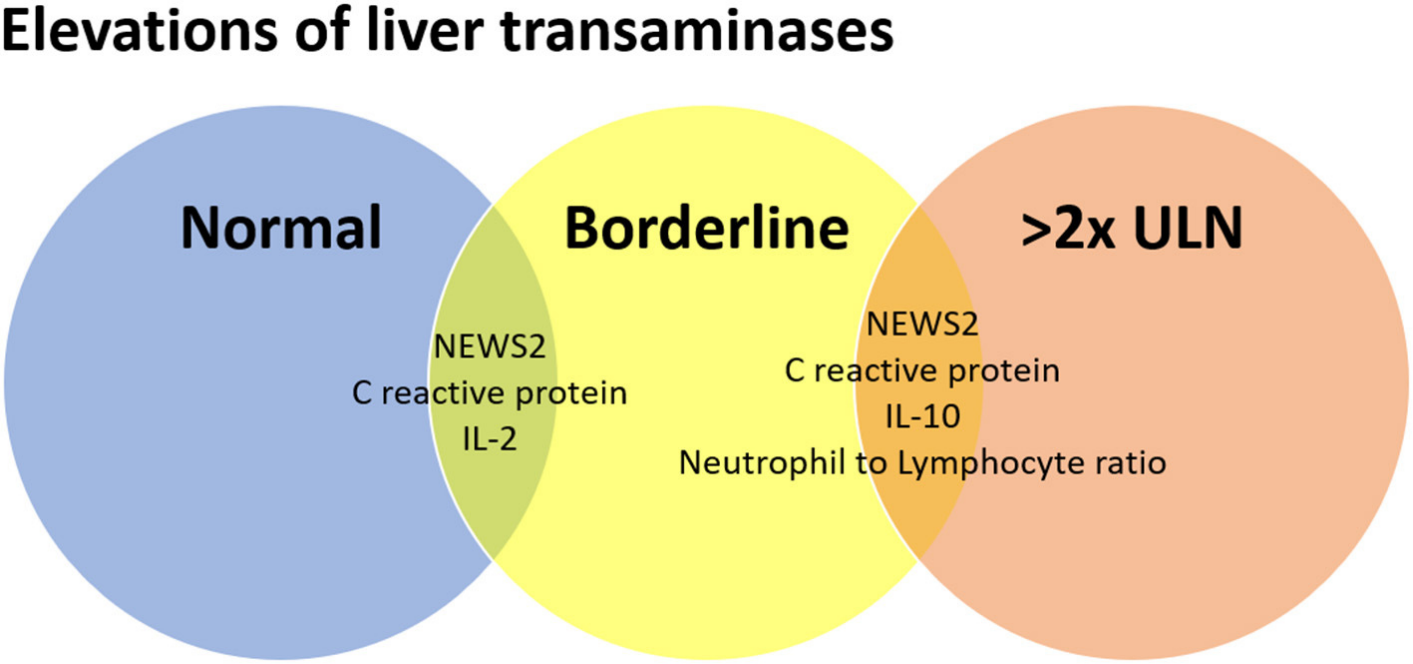
Relationship between the elevations of liver transaminases and inflammatory markers in adult patients with COVID-19.

Table 2 shows the patterns of liver transaminase tests. One hundred and sixteen (55.5%) participants presented normal liver transaminase tests on hospital admission, whereas 55 (26.3%) had borderline elevations, and 38 (18.2%) had greater than two-fold elevations. The groups were rather similar, except for NEWS2 score and disease severity on hospital admission, need for ICU admission during hospitalization, serum levels of IL-2, IL-10, and C-reactive protein, and Neutrophil to Lymphocyte ratio. Pairwise comparisons revealed significant differences between those with normal liver biochemistry tests as compared to those with greater than two-fold elevations for NEWS2 score and disease severity on hospital admission, serum levels of IL-10 and C-reactive protein, and Neutrophil to Lymphocyte ratio. The results for borderline elevations were rather similar to those with normal liver transaminase tests, except for NEWS2 score and disease severity on hospital admission, serum levels of IL-2 and C-reactive protein. These results can be seen in Figure 1, as an ongoing process toward higher severity of illness scores and inflammation.

**Table 2:** Characteristics of liver transaminase tests elevations.

**Feature**	**Normal** **(*n* = 116)**	**Borderline** **(*n* = 55)**	**>2x ULN** **(*n* = 38)**	***p***
Age, years	60.5 [44, 76.5]	57 [44, 68]	55.5 [45, 71]	0.6
Female sex	55 (47.4)	23 (42.8)	14 (36.8)	0.4
Charlson Comorbidity Index	2 [0, 4]	2 [0, 4]	2 [0, 3]	0.6
NEWS2 on admission	3 [2, 5]	5 [3, 7]	5 [4, 7]	0.001^a^^b^
Disease severity				0.003
Mild	18 (15.5)	4 (7.5)	0 (0)	
Moderate	54 (46.6)	20 (36.4)	13 (34.2)	
Severe	44 (37.9)	31 (56.4)	25 (65.8)	
Time from symptom onset to hospital admission, days	7 [4, 10]	7 [5, 10.5]	7 [5, 8]	0.3
Length of hospital stay, days	7 [1, 14]	8 [4, 17]	9 [5, 19]	0.07
Need for ICU admission during hospitalization	36 (31)	24 (43.6)	21 (55.3)	0.01
30-day mortality	8 (6.9)	9 (16.4)	6 (15.8)	0.08
Admission to ICU or death	39 (33.6)	27 (49.1)	23 (60.5)	0.007
Inflammatory markers				
Interleukin-2, pg/ml	13.8 [12.7, 14.9]	14.5 [13.6, 15.4]	14.2 [13.5, 14.9]	0.01^b^
Interleukin-4, pg/ml	19.8 [19, 20.8]	20.1 [19.6, 20.9]	20 [19.2, 21.8]	0.3
Interleukin-6, pg/ml	35.3 [21.5, 99.8]	42.9 [27, 111.7]	41.7 [30.4, 114.8]	0.06
Interleukin-10, pg/ml	39.7 [36.7, 46.9]	41.9 [37.6, 49.8]	44.9 [39.2, 55.9]	0.03^a^
Tumor necrosis factor α, pg/ml	13.3 [12.6, 14.7]	13.3 [12.9, 14.6]	13.3 [12.8, 14.6]	0.6
Interferon γ, pg/ml	14.9 [13.9, 17.1]	15.1 [14.1, 17.8]	16.1 [13.6, 18.6]	0.5
IL-6/IL-10 ratio	0.9 [0.6, 1.6]	1.0 [0.7, 2.1]	0.9 [0.7, 1.8]	0.1
C-reactive protein, mg/dl	6.7 [0.9, 13.5]	11.8 [4.8, 21.1]	23.6 [7.7, 17.2]	<0.001^a^^b^
N/L ratio	4.7 [2.8, 8.4]	6.3 [3.6, 10]	6.7 [4.4, 10.6]	0.02^a^

*Continuous data are presented as median [1st, 3rd quartile]. Categorical variables are presented as counts (percentages). Post-hoc significant (p < 0.05) pairwise comparisons*:

a *normal vs. > 2-fold elevation,*

b *normal vs. borderline. N/L ratio, neutrophil to lymphocyte ratio*.

**Figure 2:**
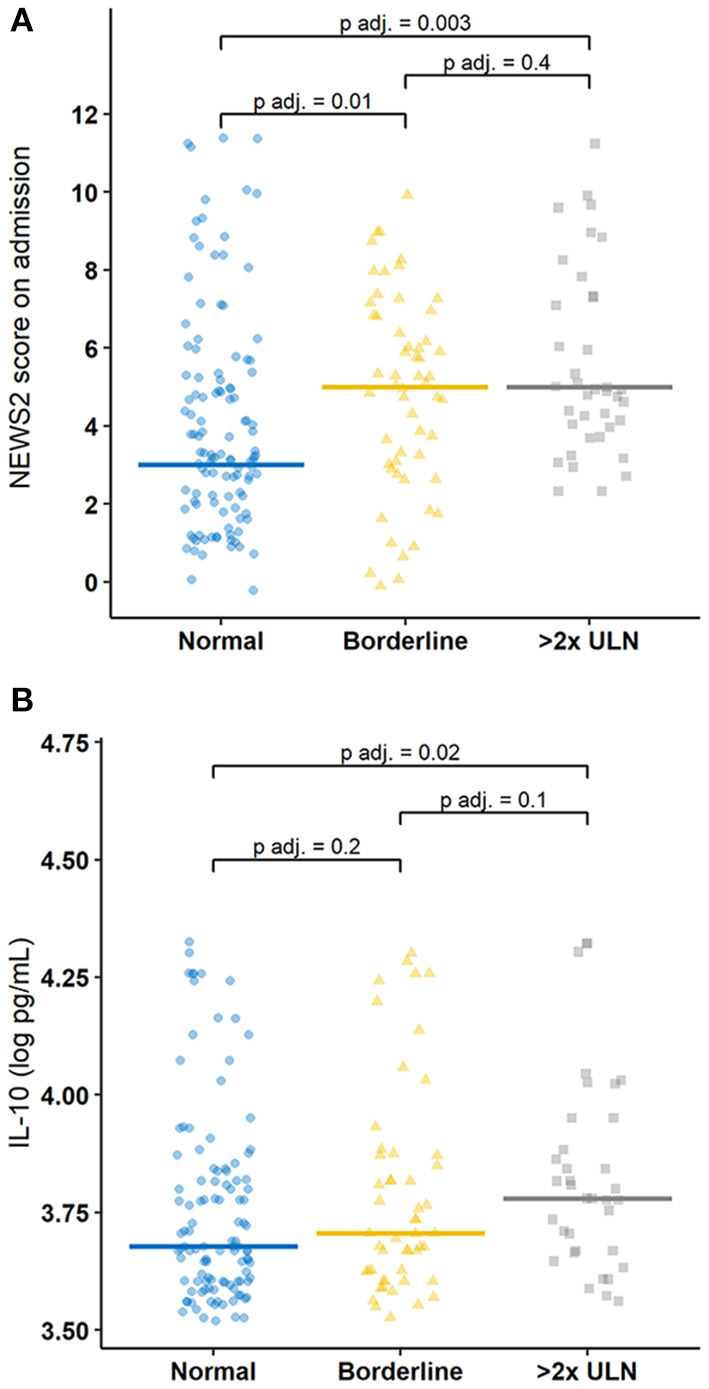
Jitter plot of the distribution of **(A)** NEWS2 score on hospital admission and **(B)** IL-10 (log pg/ml) measurements according to the liver transaminase group.

Figure 2A shows the distribution of NEWS2 score on hospital admission in each group. Patients with normal liver transaminase elevations had significantly lower NEWS2 scores on hospital admission as compared to the other two groups. Figure 2B shows that participants with liver transaminase elevations >2x ULN had significantly higher serum levels of IL-10 (log pg/ml) as compared to those with normal liver transaminase levels.

## Discussion

Since the publication of the first series of COVID-19 cases, hepatic involvement has been demonstrated through the elevation of liver enzymes, which may occur through a direct target of SARS-CoV-2 or secondary to the systematic changes caused by viral infection (22). In this study, we showed that disease severity and inflammatory markers are associated with progressive elevations in liver transaminase levels in adult patients with COVID-19. Moreover, plasma levels of IL-2 were associated with elevations exceeding the ULN range but less than two-fold the ULN, while Neutrophil to Lymphocyte ratio and IL-10 were associated with an increase of at least two-fold the ULN.

Although there are several mechanisms by which SARS-CoV-2 leads to liver damage, four have been considered more frequently: (1) direct cytopathic effect, (2) secondary to systemic inflammation, (3) an exacerbation of preexisting liver disease, and (4) drug-induced toxicity from drugs used in treatment. First, a direct cytopathic effect of the virus, although unlikely, should be considered, given the presence of ACE-2 receptors in hepatocytes (23). However, the second mechanism seems to be more likely to occur. It is a collateral damage of an unregulated immune response in severe cases in which there is a massive release of inflammatory cytokines that end up causing liver damage (8, 10, 14). Cases of acute liver decompensation have been reported in patients with COVID-19 and pre-existing liver disease, particularly in cirrhosis and alcohol-related liver disease (12, 24, 25). Drug-induced toxicity from drugs used in treatment has also been considered (17). Numerous drugs have been used worldwide in order to treat COVID-19 patients and hepatotoxicity has already been proven to be a potential side effect of a number of these drugs (11, 23, 26, 27).

Here, we hypothesized that liver damage reflects the ongoing dynamic between viral infection and the immune response. First, we demonstrated that the elevations in liver transaminases were directly related to disease severity, showing their association with the NEWS2 score at admission and the greater need for ICU or death, which corroborates with previous studies that correlated the elevation of liver transaminases with disease severity (6, 10, 11, 14, 16, 17, 28, 29). Specifically, Saini et al. (29) have shown that 21% of patients with COVID-19 and normal liver enzymes required ICU, as compared to 37 and 52% among those with raised liver enzymes and liver injury, respectively.

Then, we showed that higher levels of IL-2 and CRP were associated with elevations exceeding the ULN range but less than two-fold the ULN. IL-2 plays a central role in the modulation and expression of cell receptors of several other cytokines and transcription factors, promoting or inhibiting cytokine cascades that correlate with the proliferation of CD4+ and CD8+ T cells and with the activity of natural killer cells (30, 31). In patients with COVID-19 hospitalized due to hypoxemia, IL-2 appears at higher levels although studies did not demonstrate a direct correlation with a worse outcome (32–34). Therefore, considering the fundamental role of IL-2 in the activation of T cells, and that the increase in their plasma levels reflects the response of the effector cells to SARS-CoV-2, we hypothesize that the elevations of liver transaminases exceeding the interval of ULN, but less than two times ULN, may be associated with viral clearance of liver cells. Of note, a positive correlation between plasma CRP levels and elevated liver enzymes has been described by others (11, 29).

In line with this assumption, our study evidenced that Neutrophil to Lymphocyte ratio and IL-10 are associated with an increase of at least two-fold the ULN in liver transaminases, which may suggest the effect of hyperinflammation leading to liver damage (17). Moreover, a persistent lymphopenia has been described as a marker of disease severity in COVID-19 since the description of the first series of cases. Qin et al. (35) demonstrated the presence of lymphopenia, higher infection-related biomarkers (procalcitonin, erythrocyte sedimentation rate, serum ferritin, and CRP) and elevation of several inflammatory cytokines (IL-2R, IL-6, IL-8, IL-10, and TNF-α) in severe cases of COVID-19 as compared with non-severe ones.

However, immune response is not an isolated event, but rather an ongoing series of events. It has been demonstrated that a progressive drop in lymphocyte count leads to the progressive elevation of IL-10 levels, which serves as a regulator of inflammatory cytokines and an enhancer of B cell proliferation (36–38). Accordingly, studies have linked higher levels of IL-10 in patients with COVID-19 to increased production of other systemic inflammatory cytokines, which can contribute to the severity of the disease (36, 39). Thus, IL-10 appears to be a key cytokine in the inflammatory process related to COVID-19 and to correlate IL-10 levels with other inflammatory parameters may contribute to the understanding of how this cytokine storm can lead to damage in other organs, like the liver.

This work has some limitations that include its cross-sectional, single-center nature that avoid predictive causal result inferences. Moreover, the levels of liver enzymatic and inflammatory parameters were recorded once at the moment of admission, whereas consecutive measurements would have given a better idea of the dynamics between immune response and liver transaminases elevations. Also, despite the low prevalence for alcohol consumption, chronic viral hepatitis, cirrhosis, and the presence of hepatic steatosis was not evaluated in the study population. In addition, the low prevalence of each comorbidity assessed in this study made it impossible to analyze its individual impact on the inflammatory profile and disease severity. Yet, taken together, our results reinforce the importance to analyze the levels of liver transaminases in patients with COVID-19 as a complementary marker of disease severity. Moreover, it may also reflect the ongoing dynamic between viral infection and the immune response.

In brief, liver transaminases are complementary markers of disease severity in patients with COVID-19. IL-2 emerges as a potential marker for borderline transaminase elevations, whereas IL-10 is mainly associated with moderate to severe transaminase elevations. These associations reflect the continuous dynamics interplay between viral infection and the immune response, and its consequences.

## Data Availability Statement

The raw data supporting the conclusions of this article will be made available by the authors, without undue reservation.

## Ethics Statement

The studies involving human participants were reviewed and approved by UFSCar's Research Ethics Committee (Number: 30184220.8.0000.5504). The patients/participants provided their written informed consent to participate in this study.

## Author Contributions

RL: investigation, performed the experiments, and writing. NB: investigation and data curation. SC: investigation, data curation, and writing. MC and FA: writing, original draft, and performed the experiments. HP-J: conceptualization, methodology, investigation, writing, and original draft. All authors read and approved the final version.

## Conflict of Interest

The authors declare that the research was conducted in the absence of any commercial or financial relationships that could be construed as a potential conflict of interest.
